# Application of human-in-the-loop hybrid augmented intelligence approach in security inspection system

**DOI:** 10.3389/frai.2025.1518850

**Published:** 2025-01-22

**Authors:** Ying Huang, XiaoKan Wang, Yong Zhang, Li Chen, HongJi Zhang

**Affiliations:** ^1^The First Research Institute of the Ministry of Public Security of P.R.C., Beijing, China; ^2^Beijing Zhongdun Anmin Analysis Technology Co., Ltd., Beijing, China

**Keywords:** human machine collaboration, human-in-the-loop, hybrid-augmented intelligence, security inspection, contraband detection, workload, work efficiency, safety margin

## Abstract

A security inspection system exemplifies human-machine collaboration, and enhancing its safety and reliability through advanced technology remains a key research priority. While deep learning has incrementally improved the autonomous capabilities of security inspection equipment for automatic contraband detection, a gap persists between current technological capabilities and practical implementation. Recognizing that humans excel at learning, reasoning, and collaborating, while artificial intelligence offers normative, repeatable, and logical processing, we propose a human-in-the-loop hybrid augmented intelligence approach. This approach addresses the practical needs of security inspection systems by introducing a hybrid decision-making method that leverages two distinct strategies: “Reject-priority” and “Clear-priority.” These strategies play complementary roles in bolstering the decision-making process’s overall performance. Comparative experiments on a dataset from a specific security inspection site confirmed the hybrid method’s effectiveness, drawing several conclusions. This “Hybrid decision-making” method not only enhances risk perception, thereby widening the safety margin of the security inspection system, but also reduces the need for human labor, leading to increased efficiency and reduced labor costs. Additionally, it is less time-consuming, further improving the system’s overall efficiency. By integrating human and machine intelligence, this method significantly boosts decision-making effectiveness. Tailored to their unique characteristics, the method based on “Reject-priority” strategy is particularly well-suited for security inspection scenarios that demand stringent safety protocols, while the “Clear-priority” method is ideal for scenarios with high-volume traffic flow, where efficiency is paramount. As the volume of collected data grows, this approach will enable seamless adaptation of the method to evolving application needs.

## 1 Introduction

The security inspection system plays a crucial role in the public social security framework. Its primary function is to identify and intercept potentially dangerous and prohibited items that might be introduced into the security area with malicious intent, thus preventing security threats. Any errors in the security inspection process could compromise the entire system, placing it in a vulnerable position. In the event of a terrorist attack, such failures could lead to severe consequences, including loss of life and significant societal impacts. Consequently, researchers are dedicated to continuously improving the safety and reliability of the security inspection system by integrating advanced technological solutions to enhance its capability to detect hazardous individuals and objects.

As a mission-critical system, a security inspection system is a paradigmatic human-machine collaboration system. In this setup, the security inspection device captures perceptual information (e.g., visual images, auditory sounds) by scanning passengers’ luggage or bodies, and assists the security inspector in determining whether it is safe, resulting in decisions of either “clear for passage” (“Clear”) or “reject for passage” (“Reject”). This type of system places high demands on the security inspector’s work attitude and abilities. During inspections, the security inspector must maintain sustained attention over extended periods to detect occasional security risks. Continuous vigilance is essential, emphasizing the need for prolonged, unwavering attentiveness. Detecting risks accurately and swiftly in a cluttered environment presents a significant challenge. Additionally, the unpredictable nature of potential threats can significantly impact security inspectors’ attitudes, potentially leading to decreased performance and resulting in an elevated missed detection rate (Tian, 2011) directly. Therefore, enhancing the safety and reliability of the security inspection system through advanced technological solutions remains an ongoing research priority. As early as 2001, the U.S. Department of Transportation released a survey of airport security inspections. The missed detection rate of contraband in U.S. airport security inspection was 68% in those years (Gerald, 2001). It underscores the significance of addressing human error in the security inspection system. For over two decades, the reliability of security inspectors has remained a persistent concern for researchers (Kierzkowski and Kisiel, 2015; Yu et al., 2017). Researchers have conducted extensive studies to analyze root causes and develop effective solutions. For instance, some investigations have examined the impact of psychological stress on performance and explored the underlying psychological factors contributing to work-related stress (Cheng and Liang, 2011). Several studies have highlighted the relatively low educational requirements and skill demands for security inspectors, coupled with high job mobility and repetition. These factors contribute to a lack of enthusiasm among young people for this profession (Tian, 2011). From an ergonomic perspective, researchers have analyzed security screening using X-ray images as a typical visual search task. They have identified key influencing factors and developed visual search training methods to enhance the performance of security inspectors (Wang and Zhang, 2008; Jin et al., 2014; Liu et al., 2017; Airport Security Screener Competency, 2013).

Increasing numbers of researchers believe that security screeners face greater psychological stress and labor intensity due to their crucial role (Wang et al., 2016). Beyond scientific visual search training, automated assistance from machines may represent another viable path to enhance security performance. Early studies revealed that employing direct cue guidance to indicate target locations can aid in training security inspectors to improve their ability to identify contraband (Goh et al., 2005). Consequently, numerous subsequent investigations have focused on leveraging artificial intelligence to augment the automatic detection capabilities of machines.

The widespread adoption of deep learning in machine vision has led to gradual improvements in the self-execution capabilities of security inspection equipment for automatic contraband detection. Researchers have developed various contraband recognition network models specifically designed for X-ray imaging (Yang, 2021; Ding et al., 2019). These models have demonstrated significant enhancements in key performance indicators on test datasets, including accuracy, recall, and detection efficiency. The advancement of machine intelligence has enabled the integration of automatic detection technologies into practical security inspection systems. Leveraging the quantifiable, durable, and uniformly standardized specifications of machine intelligence can compensate for human limitations in several aspects, thereby enhancing the safety and reliability of security inspection systems. However, this emerging research area remains in its exploratory and optimization phase. The gap between current technological capabilities and practical implementation persists due to several factors:

1. Regarding universality, the model’s generalization capacity is insufficient. Its exceptional performance in numerous experiments is limited to specific item types. The model demonstrates excellent accuracy only on test images of comparable quality to the training set. This restricts the comprehensive evaluation of these methods in practical applications.
2. In terms of practicality, achieving a balance between the model’s accuracy and computational efficiency is challenging. High-accuracy models with complex architectures are frequently employed to produce outstanding recognition results, but these come with substantial computational demands. This approach necessitates devices with high processing power, which can be resource-intensive. In the context of actual security inspection processes, computational efficiency is equally crucial as accuracy for the method to be practically useful. Simply increasing costs to boost computing power is not an optimal strategy for real-world applications.
3. From an ergonomic perspective, replacing human decision-making with automated models may introduce risks and hidden dangers. Some researchers have commented on the use of automatic machine detection to assist security inspectors in making decisions. For instance, this highly human-dependent visual inspection task need to explore what can be partially replaced by a machine firstly (Goh et al., 2005). However, there is still a significant gap between the capabilities of machines and humans. This disparity can lead to excessive false positives, potentially interfering with security inspectors and affecting the efficiency of the security inspection system. Additionally, if security inspectors rely heavily on machines for decision-making tasks, it may cause a decline in their alertness, compromising the safety margin of the system. Furthermore, for novice security inspectors, relying on machine-assisted functions may weaken their ability to acquire knowledge and hinder the enhancement of their operational capabilities.

Given the growing reliance on human-machine collaboration in security inspection, machine automatic detection serves as a valuable decision-making tool to support security inspectors. While its potential benefits to the security system are promising, but further research is needed to comprehensive demonstrate its reliability and effectiveness.

## 2 Related works

### 2.1 Application of artificial intelligence in security inspection

Over the years, contraband identification based on deep learning technology has evolved and been implemented in practical applications. Recent research focuses on addressing challenges such as limited training samples, item occlusion, and complex background interference. Several researchers have proposed improved models, including YOLO-CID, Material-Aware Path Aggregation Network, and FSVM. These models have been tested on publicly available X-ray baggage image datasets.

YOLO-CID (Gan et al., 2023) is an enhanced variant of the classic YOLO object detection algorithm, designed to improve detection performance in complex scenarios by incorporating contextual information. By integrating contextual cues, YOLO-CID can better interpret image content, particularly when dealing with intricate backgrounds, occluded targets, blurry edges, or small-sized objects. Experimental results on the PIDray public dataset demonstrate YOLO-CID’s efficacy, having an average accuracy of 82.7% and a recall rate of 81.2%, representing increases of 4.9 and 3.2% over YOLOv7, respectively. On the CLCXray dataset, YOLO-CID achieves an mAP of 80.2%. Furthermore, it enables real-time detection at 43 frames per second in actual scenarios.

Material-Aware Path Aggregation Network (Xiang et al., 2023) is an enhanced variant of the YoloX-based material-aware path aggregation network, specifically designed to address the challenges of multi-scale and occlusion in X-ray contraband detection. This approach effectively mitigates the obscuring problem by emphasizing distinctive material characteristics. The network also implements a shape-decoupled SIoU (SD-SIoU) loss function, which balances the effects of long-short sides by integrating a category decoupling module and a long-short side decoupling module. This innovative combination of techniques enables the detection of challenging samples in extreme cases, such as small objects and occluded items. Experimental results on the OPIXray and SIXray datasets demonstrate the effectiveness of this approach, with average accuracies of 92.65 and 91.31%, respectively. The model’s parameter scale is 109.46 M, and its computational requirement is 256.86 G.

FSVM (Fang et al., 2023) is a contraband detection model that leverages minimal sample support vector machine constraints to achieve remarkable performance with limited labeled data. This approach is designed to detect contraband using only a handful of annotated samples, making it particularly effective for scenarios with scarce training data. To generate an informative embedded space, the model incorporates an SVM-embedded module for end-to-end training. This module allows for the propagation of oversight information from the fine-tuning phase back to earlier layers, enabling the model to learn from subtle patterns in the data. For training, a subset of the SIXray dataset should be selected, focusing on the desired small sample category. The method demonstrates superior performance compared to other approaches in both 10-shot and 30-shot experiments.

Xu et al. (2023) introduce innovative concepts of whole-process feature fusion and local–global semantic dependency interaction, aiming to enhance the automatic detection of prohibited items. They conducted experiments on the challenging Security Inspection X-ray (SIXray), Occluded Prohibited Items X-ray (OPIXray), Cutters and Liquid Containers X-ray (CLCXray), and Prohibited Item Detection X-ray (PIDray) datasets. The results demonstrate that the PIXDet detector family achieves significant detection performance, with mean average precision (mAP) scores of 91.2% for PIXDet-S.

An end-to-end weakly supervised correction (WSC) method with three modules for denoising and rectifying ambiguous labels is proposed. Experimental validations show that WSC increases the average precision (AP) by 3.3 and 4.5% on the EDXray and PIDray datasets (Wang et al., 2024).

Wang et al. propose an efficient background learning (EBL) method with three modules: mixed foreground and background learning (MFB), hierarchical balanced hard negative example (HBHE) sampler and prime background mining with voting (PBMV). Experiments show that EBL can reduce false positives while maintaining high recall. When applied to Faster R-CNN, AP50 increases by 5.8% on benchmark X-ray datasets, including 2.3% on OPIXray and 3.7% on SIXray (Wang et al., 2023).

Ren et al. (2022) present LightRay, a lightweight object detection framework that builds upon the YOLOv4 algorithm. The experimental results show that the mAP of the lightRay model is 87.28% on the SIXray data set, while the FLOPs of the model are reduced to the original 1/5, and Params is reduced to 1/3 of the original. In addition, some ablation experiments confirm the ability of the LightRay model the detection of prohibited items with small sizes.

### 2.2 Human-in-the-loop hybrid-augmented intelligence method

As artificial intelligence enters the era of large-scale application, inherent challenges in specific scenarios are gradually surfacing. This has prompted many researchers to explore the interplay between artificial intelligence and human cognition. Some argue that machine learning struggles to comprehend practical environments, particularly when dealing with incomplete information and complex spatiotemporal tasks. There exists a significant gap between machine learning algorithms and human brain function in processing ambiguous situations and nuanced contextual information. The human brain’s understanding of non-cognitive factors relies heavily on intuition, which can be influenced by experience and long-term knowledge accumulation. Despite the availability of vast or unlimited data resources for artificial intelligence systems, human intervention remains essential in intelligent systems. This is particularly evident in critical application domains such as industrial risk management, medical diagnostics, and criminal justice systems. To mitigate the risks and potential harm associated with artificial intelligence, addressing its limitations is a critical challenge for its advanced development. Therefore, the future of artificial intelligence should not be an independent, isolated, self-circulating academic system, but a part of human evolution. The rational and efficient use of artificial intelligence can promote value innovation and enhance the capabilities of humans and machines.

Studies have shown that humans are better at learning, reasoning, collaborating and other advanced intelligence activities. While artificial intelligence is normative, repeatable and logical. Repetitive work does not reduce the efficiency or accuracy of the machine, so artificial intelligence is better at handling discrete tasks rather than discovering or breaking rules on its own. Artificial intelligence and human intelligence each have advantages and are highly complementary. Researchers believe that introducing human supervision, human-computer interaction and manual verification into artificial intelligence systems increases the confidence of systems, build human-in-the-loop hybrid augmented intelligence and better utilize human knowledge. In hybrid augmented intelligence systems, human intelligence is a component within the loop. The predictions and interventions by humans enhance the accuracy and credibility of the system. By integrating human perception and cognitive abilities with machine computation and storage capabilities, the system can handle large-scale, incomplete, and unstructured knowledge information. To prevent the risks of out of control associated with artificial intelligence, manual intervention will be implemented when the confidence of machine decision-making is low, the system’s knowledge base will also be automatically updated subsequently. In fact, the hybrid learning model of intelligence can greatly expand the scale and efficiency of tasks that humans can accomplish (Zheng et al., 2017).

Leveraging the concept of hybrid augmented intelligence, Researchers have already embarked on cutting-edge research across various domains. In the military domain, for instance, where the environment is characterized by high complexity, blurred boundaries, intense confrontation, need for rapid real-time responses, and sparse data samples. It is believed that effective military applications require intricate human-computer interactions and interdependencies that neither simple artificial intelligence nor human intelligence alone can achieve optimally. Thus, the integration of human and machine intelligence represents a pivotal direction for advancement (Zhang and Zhang, 2021; Cheng et al., 2020).

In the realm of intelligent power system management, the proliferation of data accessible through an open intelligent analysis platform presents significant challenges. Traditional intelligent analysis methods struggle with the complexity of data acquisition settings and are prone to lengthy, time-consuming model creation and analysis processes. These inefficiencies hinder the system’s ability to meet user demands promptly, which is detrimental to the seamless operation of various application functions. By integrating human-computer intelligence technology, the system can semantically interpret user requests to define analytical objectives, enabling precise knowledge mapping. Guided by this knowledge, the system selectively collects the necessary data for analysis, obtaining a relevant data subset that avoids overwhelming the system with excessive data scope. This approach not only alleviates the pressure on system analysis but also accelerates the analysis process, thereby reducing the operational complexity of the intelligent analysis platform (Lin, 2020).

In intelligent transportation systems, merely enhancing the intelligence of vehicles and infrastructure to alleviate traffic congestion is insufficient, as it overlooks the critical human factors. While intelligent transportation aims to minimize the driver’s role, effectively liberating them from conventional driving, this approach often results in a system with suboptimal reliability and low safety margin due to the absence of driver input in decision-making processes. This can lead to inadequate responses to complex roadway scenarios. Integrating human decision-making into the system’s control loop creates a human-in-the-loop hybrid augmented intelligence traffic system. This integration leverages the strengths of both human logic and the execution capabilities of intelligent vehicles, forming a complementary and cohesive system. By adopting a human-vehicle integration model, the system can achieve faster response times. Human intelligence compensates for the limitations of autonomous vehicle operation, thereby enhancing the control performance of the entire intelligent traffic system. This approach not only improves the reliability and flexibility but also significantly bolsters the safety of intelligent transportation systems (Qian et al., 2019).

In the field of industrial robotics, researchers have introduced hybrid augmented intelligence to the research of grab algorithms in collaborative robots (Fu et al., 2019). At present, the ability derived from deep learning training with limited model data is the most basic capability, which can only achieve very limited grabbing, while actual scenarios vary greatly. Robots that rely solely on model data collected or generated by humans are unlikely to achieve satisfactory outcomes, particularly in dynamic environments where the cost to humans is high and the applicability is limited. To address the issue of adapting to varying environments while in motion, researchers have integrated a human-in-the-loop cognitive input model. In the event of a failed grabbing attempt by the robot (indicated by low confidence), human intervention is employed. This allows the robot to construct a knowledge base informed by human strategies and to engage in self-learning, thereby equipping it to handle unfamiliar and complex work scenarios effectively.

### 2.3 Hybrid-augmented intelligence in security systems

The primary function of a security system is fundamentally a visual cognitive task. Security inspectors visually examine X-ray images of luggage and combine prior knowledge to identify the presence of prohibited items. However, X-ray images are perspective representations, and the overlapping and projection distortions between items in luggage significantly complicate cognitive processing. This complexity renders it challenging for intelligent algorithms to independently execute the intricate process of image comprehension, reasoning, and decision-making, especially when faced with limited training data.

Cao et al. (2019) presented a human-in-the-loop framework for luggage inspection. This framework employs a deep-learning algorithm to detect contraband in X-ray images of luggage, opting for manual review when the algorithm is uncertain about the safety of an item. The benefits of this inspection process include the ability to capture new sample images for incremental training of the detection model and the enhancement of detection intelligence through human-computer collaboration. Preliminary experimental results indicate that the human-in-the-loop approach, which combines the cognitive abilities of human inspectors with the capabilities of intelligent algorithms, significantly boosts the accuracy of contraband detection in baggage security screening.

However, Cao’s research focused on enhancing deep learning models’ capabilities, potentially overlooking the significance of human-in-the-loop approaches. This limitation may result in suboptimal performance in certain security screening environments, leading to less efficient security inspections.

Based on the aforementioned issues, in conjunction with practical scenarios in the security inspection, this paper concentrates on the application of human-in-the-loop hybrid augmented intelligence methods in security inspection systems. It analyzes typical security inspection practical requirements, which the recall rate of contraband recognition is the primary metric of concern. A higher recall rate indicates a greater safety margin, suggesting that the security inspection system is more reliable and secure. Therefore, the primary objective of hybrid augmented intelligence is to enhance the recall rate of contraband recognition. By introducing machine intelligence for automatic contraband detection, it alleviates part of the workload for human inspectors, thereby reducing the risk of decreased vigilance due to fatigue. At the same time, leveraging the computational advantages of machine intelligence can improve operational efficiency in low-complexity, high-repetition interception decisions, and assist human inspector to check for omissions. Thereby increasing the safety margin of security inspection system.

## 3 Methods

### 3.1 Fundamental consideration for application of method in security inspection

While most technical literature focuses on improving the performance of contraband recognition using deep learning models (Richard et al., 2021), there is a lack of guidance on how to assess and evaluate methods in practical scenarios. To address this gap, we introduce the concept of appropriateness, which examines how well an intelligence method aligns with the specific context of security inspection.

Analyzing the core concerns of security inspection, contraband identification emerges as the most critical ability. Higher “Contraband recall rates” correlate directly with increased safety margins, indicating a more reliable security inspection system. Consequently, the primary objective of hybrid augmented intelligence is to enhance contraband detection accuracy by introducing machine intelligence to automate contraband identification and reduce manual labor. This approach aims to decrease errors resulting from fatigue and distraction. Furthermore, leveraging high-computing capabilities, machine intelligence can improve the effectiveness of intercepting low-complexity and high-repetitive common contraband.

### 3.2 Method description

The proposed human-in-the-loop hybrid augmented intelligence approach encompasses a novel hybrid decision-making method. This method comprises two sequential steps:

Step 1: Initial Machine Intelligence Identification.

Machine intelligence assumes the initial decision-making role, leveraging its strengths in identifying low-complexity and high-repetitive common contraband at high speeds. The process begins with undifferentiated global detection across entire X-ray images and preliminary screening.

Step 2: Human-Informed Secondary Screening.

Based on optimization objectives, select images for human review through various strategies. Conduct secondary screening based on preliminary screening results, utilizing human expertise in identifying high-complexity and rare contraband. The final decision is formed by merging results from both preliminary and secondary screenings in sequence.

Two distinct strategies correspond to different optimization objectives: “Reject-priority” and “Clear-priority.” Flowcharts for both strategies are provided below.

#### 3.2.1 “Reject-priority” strategy

**Figure 1:**
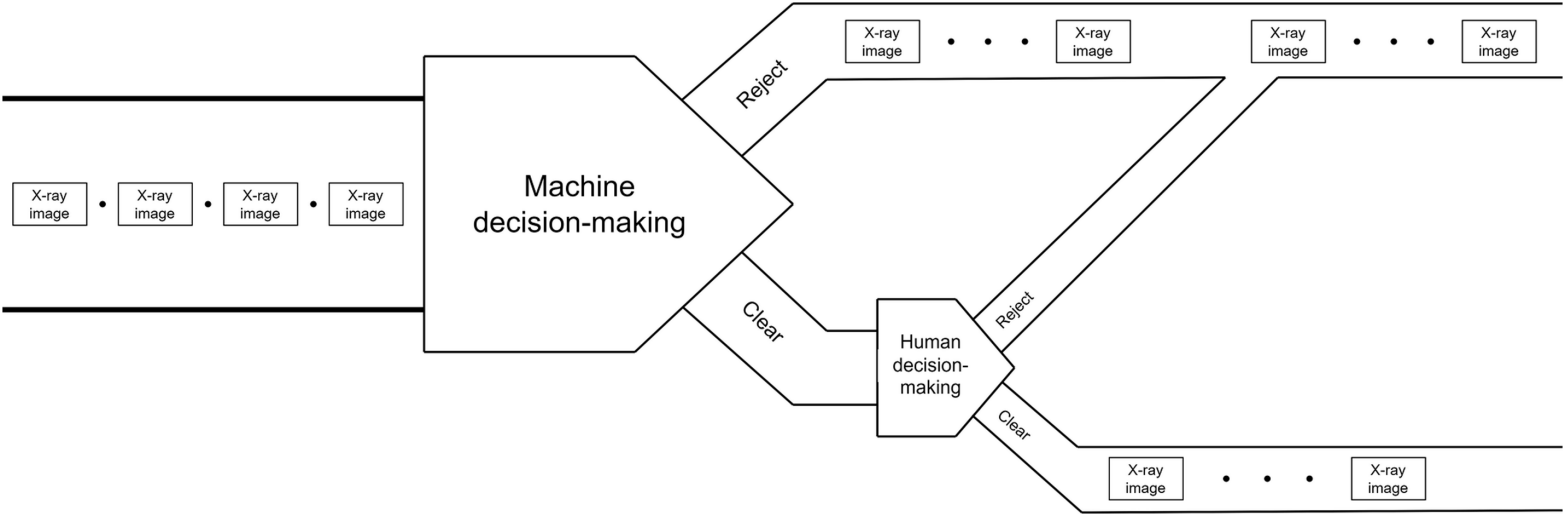
“Reject-priority” strategy.

**Figure 2:**
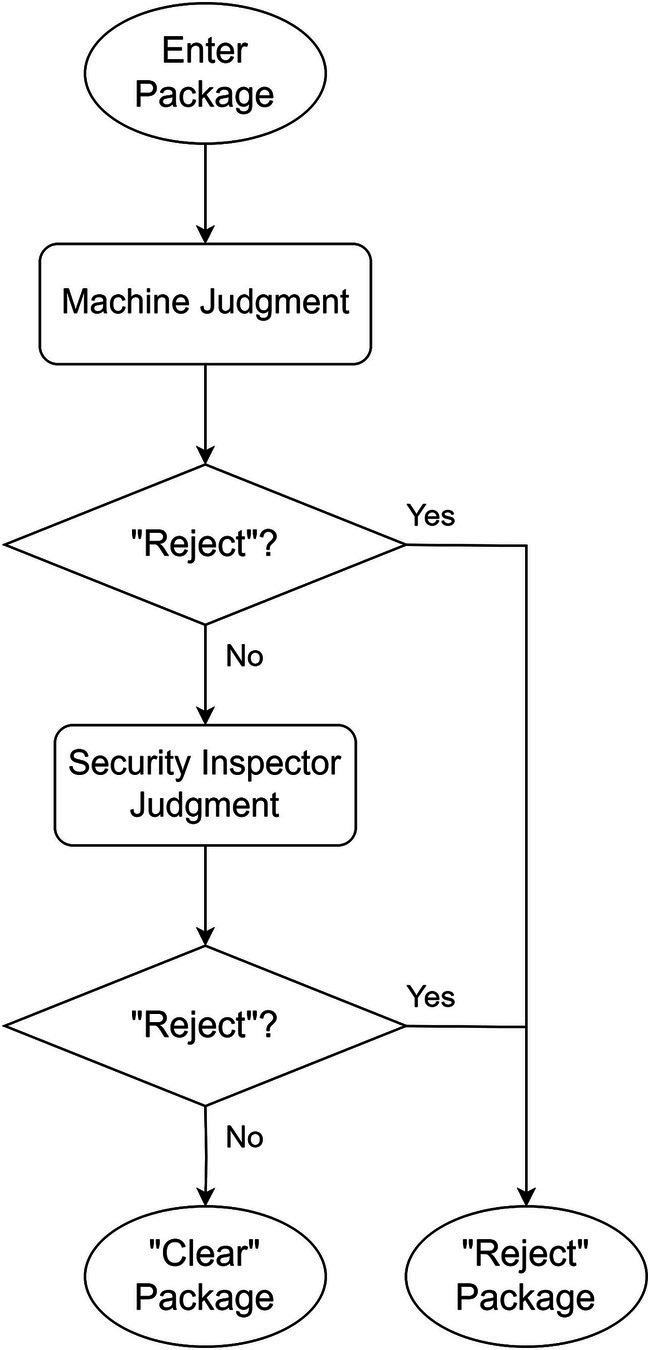
Flowchart of “Reject-priority” strategy.

“Reject-priority” means believing in the machine’s preliminary decision of “Reject,” decisions of “Clear” is pushed to human doing secondary decision. Through the human decision-making process, machine’s wrong decision of “Clear” (i.e., Missed detection) is corrected by human as the final decision of “Reject.” Only the decisions of “Clear” confirmed by human can be the final decision of “Clear.” The schematic diagram of strategy is shown in Figures 1, 2 shows the flowchart of the “Reject-priority” strategy.

The “Reject-priority” strategy operates as follows:

Machine intelligence initiates the decision-making process by screening X-ray images automatically.

If the machine determines “Reject,” this decision stands as the final outcome.

If the machine suggests “Clear,” the image is flagged for human review.

Upon human examination, incorrect “Clear” decisions (Missed detections) are corrected to “Reject.”

Only images confirmed as “Clear” by human inspection become the final “Clear” decision.

#### 3.2.2 “Clear-priority” strategy

**Figure 3:**
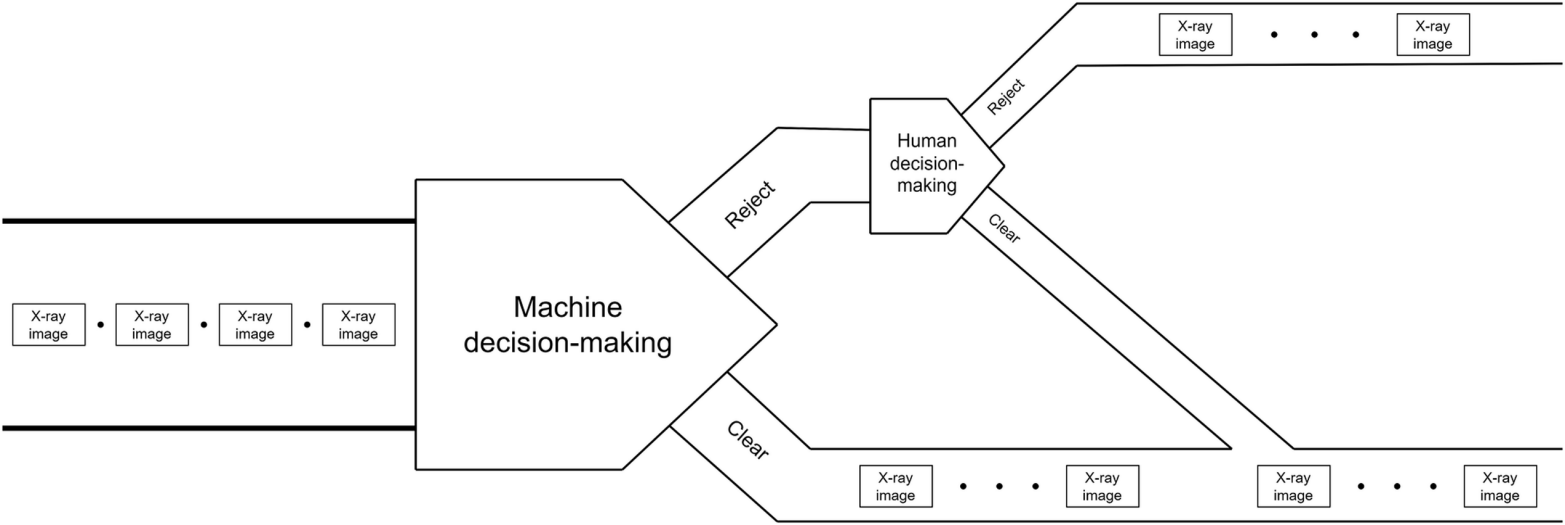
“Clear-priority” strategy.

**Figure 4:**
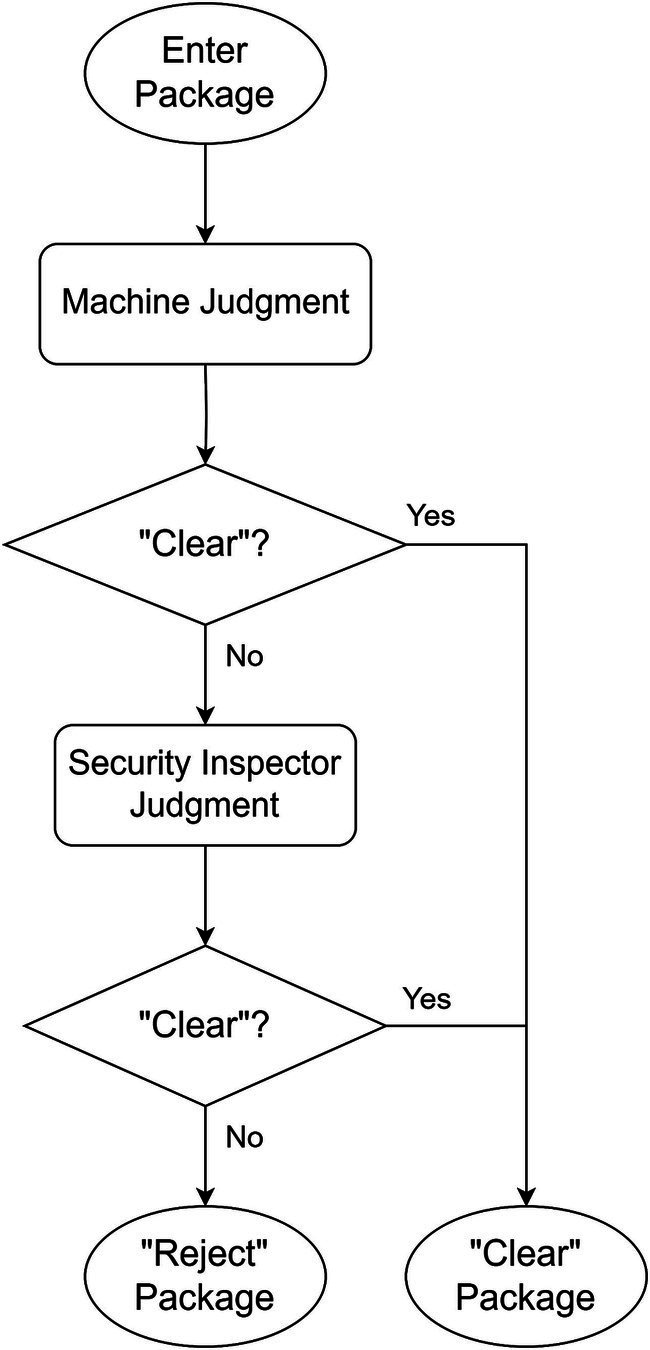
Flowchart of “Clear-priority” strategy.

The schematic diagram of strategy is shown in Figures 3, 4 shows the flowchart of the “Reject-priority” strategy.

The “Clear-priority” strategy operates as follows:

Machine intelligence initiates the decision-making process by examining X-ray images.

If the machine determines “Clear,” this decision stands as the final outcome.

If the machine suggests “Reject,” the image is flagged for human review.

Upon human examination, incorrect “Reject” decisions (False alarms) are corrected to “Clear.”

Only images confirmed as “Reject” by human inspection become the final “Reject” decision.

This approach ensures that potential threats are not overlooked while minimizing unnecessary human intervention for clear cases.

#### 3.2.3 Model for contraband detection

We previously proposed an XMC R-CNN model for contraband detection (Zhang et al., 2020). Most deep learning-based contraband detection algorithms utilize RGB images, failing to leverage the rich information present in X-ray high and low energy data. Consequently, these methods often neglect to fully utilize material-specific information during detection. The challenge of contraband detection within X-ray baggage imagers remains understudied in the machine vision community due to the scarcity of publicly available X-ray image datasets. Given the complexity of this problem, detection models that incorporate X-ray high and low energy data are notably limited in the existing literature.

**Figure 5:**
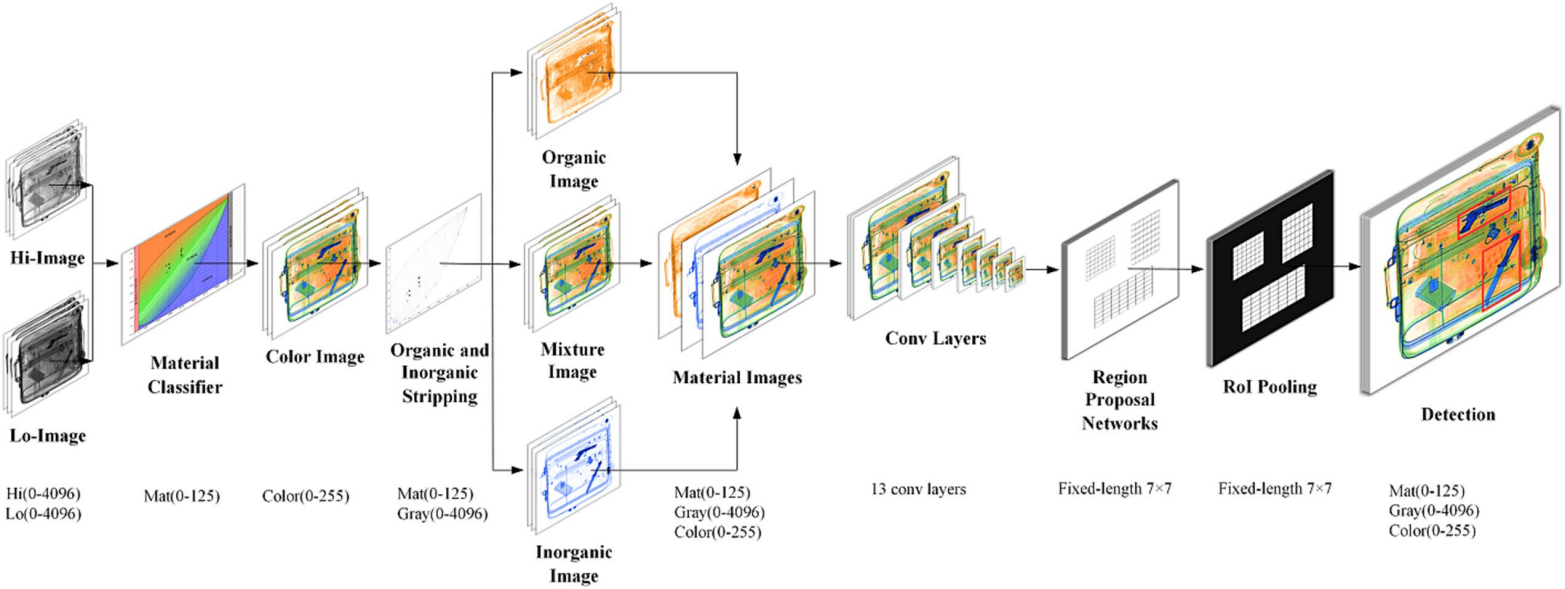
XMC R-CNN model (Zhang et al., 2020).

As shown in Figure 5, the XMC R-CNN model is composed of two modules. The Priority module is X-ray Material Classifier (XMC) that strips organic and inorganic, and the second module is the Faster R-CNN detector that uses the proposed regions. The main contribution of the XMC R-CNN model is having solved the problem of contraband detection in overlapped X-ray baggage images. The detection rate and miss rate that meet the requirements of practical scenarios are achieved. It was verified that the detection rate is greater than 95%, and the miss rate is less than 5%. In some applications, it has exceeded the level of security inspectors.

Figure 5 illustrates the XMC R-CNN model proposed by Zhang et al., comprising two key modules:

1. The Priority module: X-ray Material Classifier (XMC)

Distinguishes between organic and inorganic materials; Performs initial screening of X-ray images.

1. The Detection module: Faster R-CNN

Utilizes regions proposed by the XMC; Performs subsequent detailed analysis.

The primary contribution of the XMC R-CNN model lies in solving the complex problem of contraband detection in overlapped X-ray baggage images. Notably, it achieves detection rates exceeding 95% and miss rates below 5%, meeting practical scenario requirements. In some applications, its performance surpasses that of human security inspectors.

In practical security inspection settings, where efficient throughput is paramount, machine intelligence plays a crucial role in rapidly identifying contraband within the “human-in-the-loop hybrid augmented intelligence” framework.

Our research aims to address the practical requirements of security inspection systems. While the performance of contraband detection based on the XMC R-CNN model represents capability in ideal conditions, we acknowledge that performance in practical security inspection scenarios requires further verification. This paper does not focus on the contraband detection model itself, which is excluded from further discussion.

## 4 Validation

The evaluation objective of the hybrid augmented intelligence method is to assess its practical value for security inspection. Specifically, a dedicated security inspection dataset is utilized to validate the proposed method. The validation process primarily focuses on comparing the decision-making precision and efficiency between human-machine hybrid intelligence and human intelligence.

### 4.1 Test dataset

The dataset was collected from primary hub airport security checkpoints, providing a realistic representation of security screening processes. It comprises 33,819 X-ray baggage images captured over 11,928 min of continuous operation at high throughput levels. Unlike existing datasets (e.g., PIDray, OPIXray, SIXray, and CLCXray), the proposed dataset exhibits uneven distribution of contraband categories, potentially limiting deep learning model performance. However, it accurately reflects the practical status of on-site security inspection systems. Furthermore, the dataset incorporates actual decision outcomes from over 100 security inspectors, enabling assessment of their decision-making abilities. Consequently, this dataset is suitable for validating human-in-the-loop hybrid augmented intelligence methods.

### 4.2 Test scheme

Actual decision outcomes from security inspectors includes “Reject” or “Clear” for passage. The dataset will be sub-divided in accordance with the following steps:

- Step 1: According to the actual decision (“Reject”/“Clear” for passage) made by the security inspector, the dataset is divided into two subsets which are named “Human-reject” and “Human-clear”;
- Step 2: Utilizing the contraband detection model outlined in Section 2.2, we can ascertain whether to categorize each image in the dataset as “Reject” or “Clear.” Consequently, the dataset is bifurcated into two distinct subsets, designated as “Machine-reject” and “Machine-clear”;
- Step 3: Adopting the “Reject-priority” strategy as described in Section 3.2.1, the “Machine-reject” subset is treated as the set of decisions that require unconditional trust in the human-machine hybrid-decision process. This subset serves as the basis for the “Hybrid-reject” set. Meanwhile, the “Machine-clear” subset undergoes a secondary judgment through “Human-decision.” The secondary subset that is “Human-reject” is integrated back into the “Hybrid-reject” subset, whereas the “Human-clear” subset is recognized as the “Hybrid-clear” subset.
- Step 4: Following the “Clear-priority” strategy detailed in Section 3.2.2, the “Machine-clear” subset is deemed the set of decisions that are trusted unconditionally in the human-machine hybrid-decision framework. This subset lays the groundwork for the “Hybrid-clear” subset. In contrast, the “Machine-reject” subset is subjected to a secondary judgment through “Human-decision.” The secondary subset identified as “Human-clear” is merged into the “Hybrid-clear” subset, while the “Human-reject” subset is designated as the “hybrid-reject” subset.

### 4.3 Data analysis

#### 4.3.1 Human intelligence performance

There are 9,011 images in the “Human-reject” subset, and 24,808 images in the “Human-clear” subset. The details are shown in Table 1.

**Table 1:** Decision-making results of security inspector.

Duration (min)	11,928						
Average time consuming per image(s)	21.2						
Numbers of images	33,819						
Subset	(Human-reject) 9,011						(Human-clear) 24,808
Proportion of subset (%)	26.6						73.4
Contraband category	Power banks	Cosmetics	Liquid	Lighter	Lithium Batteries	Rare categories	
Numbers of contraband images	7,400	814	309	246	98	144	
Proportion of contraband category (%)	82.1	9.1	3.4	2.7	1.1	1.6	

“Rare categories” that only take 1.6% proportion in the “Human-reject” subset include alcohol, knives, tools, mercury, and other rare contraband. The categories that account for the majority of the dataset are power banks, cosmetics, liquid, lighter and lithium batteries. This manifest the actual scenario where routine security inspections at airports are focused on detecting these specific categories of contraband.

1. Indicators related to efficiency
  - Average time consuming per image, it represents speed of decision-making;
  - Proportion of the “Human-clear” subset, it represents the first-time pass rate.
2. Indicators related to safety margin

Proportion of the “Human-reject” subset, it represents the rate of decision that open the package for inspection during the security inspection process.

#### 4.3.2 Machine intelligence performance

The contraband detection model proposed in Section 3.2.3 is sensitive to the major categories of contraband in Table 1. There are 13,240 images in the “Human-reject” subset, and 20,579 images in the “Human-clear” subset. The details are shown in Table 2.

**Table 2:** Decision-making results of the contraband detection model.

Duration (min)	56					
Average time consuming per image(s)	0.1					
Numbers of images	33,819					
Subset	(Machine-reject) 13,240					(Machine-clear) 20,579
Proportion of subset (%)	39.2					60.9
Contraband category	Power banks	Cosmetics	Liquid	Lighter	Lithium Batteries	
Numbers of contraband images	11,241	1,101	430	374	94	
Proportion of contraband category (%)	84.9	8.3	3.3	2.8	0.7	

The detection model’s ability to identify rare contraband is less effective compared to a security inspector. Consequently, the composition of the “Machine-reject” subset differs from the “human-reject” subset. This disparity leads to varying performance levels between machine intelligence and human intelligence on the same dataset. A comprehensive comparison of efficiency-related indicators and safety margins follows in the subsequent section.

#### 4.3.3 Performance comparison between human and machine

A respected security screening expert was invited to review the images within the dataset, providing additional assessments on the decisions rendered by both the security inspectors and the contraband detection model. Utilizing these secondary judgments, the decision-making outcomes of humans and machines have been recompiled and are presented in Table 3.

**Table 3:** Reorganization of decision-making results based on secondary judgments.

Case	Description of secondary judgment	Numbers of images
1	Human and machine made consistent correct decision of “reject”	7,448
2	Human made correct decision of “reject” & Machine made incorrect decision of “clear”	339
3	Human made incorrect decision of “reject” & Machine made correct decision of “clear”	745
4	Human made correct decision of “clear” & Machine made incorrect decision of “reject”	4,473
5	Human made incorrect decision of “clear” & Machine made correct decision of “reject”	840
6	Human and machine made consistent incorrect decision of “reject”	479
7	Human and machine made consistent correct decision of “clear”	19,495
Total		33,819

It can be extendedly summarized from Table 3 as follows:

1. The total number of images that should be made decision of “reject” was the sum of cases “1,” “2” and “5,” which amounts to 7,448 + 339 + 840, totaling 8,627 images.

And the total number of images that should be made decision of “clear” was the sum of cases “3,” “4,” “6” and “7,” which amounts to 745 + 479 + 4,473 + 19,495, totaling 25,192 images.

1. Indicator representing the accuracy of “Reject” decisions was referred to as the “Contraband recall rate.” This rate was the proportion of “Correct ‘reject’ decisions” to the “Total number of cases that should be ‘rejected’“. The capacity for accurate “Reject” decisions is directly proportional to the “contraband recall rate.”

- For human decision-making, the “contraband recall rate” was

(7,448 + 339)/8,627*100% = 90.3%.

- For machine decision-making, the “contraband recall rate” was

(7,448 + 840)/8,627*100% = 96.1%.

**Comparison conclusion 1**: The machine decision-making had higher “contraband recall rate” than that of human decision-making.

1. Indicators representing the precision of “Reject” decisions could be gauged by two indicators: “Contraband Detection Precision” and “False Alarm Rate.”

The “Contraband detection precision” was the proportion of the “Correct decision of ‘reject’” to the “Total numbers of that be decided as ‘reject’”.

- For human decision-making, the “Contraband detection precision” was

(7,448 + 339)/9011*100% = 86.4%.

- For machine decision-making, the “Contraband detection precision” was

(7,448 + 840)/13240*100% = 62.6%.

The “False alarm rate” was the proportion of “Incorrect decision of ‘reject’” to the “Total numbers of that be decided as ‘reject’”. The “False alarm rate” was inversely proportional to the “Contraband detection precision.”

- For human decision-making, the “False alarm rate” was

(745 + 479)/9,011*100% = 13.6%

- For machine decision-making, the “False alarm rate” was

(479 + 4,473)/13,240*100% = 37.4%.

**Comparison conclusion 2**: Human decision-making exhibits a higher “Contraband detection precision” compared to machine decision-making. Conversely, machine decision-making results in a higher “False alarm rate” than human decision-making.

1. The safety margin can be assessed using the “Contraband missed detection rate,” which is the ratio of “Incorrect ‘clear’ decisions” to the “Total number of cases that should be ‘rejected’“. The safety margin is inversely proportional to the “Contraband missed detection rate.”

- For human decision-making, the “Contraband missed detection rate” was

840/8,627*100% = 9.7%.

- For machine decision-making, the “Contraband missed detection rate” was

339/8,627*100% = 3.9%.

**Comparison result 3:** Machine decision-making boasts a lower “Contraband missed detection rate” compared to human decision-making.

1. The indicator that represents the credibility of machine decision-making, reflecting the consistency between human and machine decisions, is known as the “Agreement rate.”

- The “Agreement rate” was the ratio of “All consistent decisions of ‘reject’ and ‘clear’” to the “Whole dataset,” (7,448 + 19,495)/33,819*100% = 79.7%.

**Comparison result 4:** The machine decision-making based on the selected contraband detection model is considered credible.

All comparative analyses of the indicators are categorized by evaluation objectives and compiled in Table 4.

**Table 4:** Indicators comparison classified by evaluation objectives.

Performance		Data resource		
Classification	Indicator	Human decision-making	Contrast relationship	Machine decision-making
Efficiency	Average time consuming per image(s)	21.2	>	0.1
	Rate of making “Clear” decision (%)	73.4	>	60.9
	Contraband detection precision (%)	86.4	>	62.6
	False alarm rate (%)	13.6	<	37.4
Safe margin	Contraband recall rate (%)	90.3	<	96.1
	Contraband missed detection rate (%)	9.7	>	3.9

Table 4 presents various viewpoints for discussion. Machine intelligence offers rapid decision-making capabilities, reducing the average processing time per image to 1/200th of human decision-making speed. This significantly enhances inspection efficiency. However, machine intelligence exhibits heightened sensitivity to common contraband, resulting in lower contraband detection accuracy and first-time pass rate compared to human intelligence. The decreased accuracy may compromise overall system efficiency.

Secondly, machine intelligence demonstrates superior contraband recall rates, potentially increasing the safety margin in security inspections. Consequently, machine intelligence presents both benefits and drawbacks for security inspection systems. To maximize its potential, practical applications should focus on effectively leveraging machine intelligence’s advantages. Evaluations of hybrid human-machine approaches should prioritize balancing improved safety margins with maintaining system efficiency.

#### 4.3.4 Performance of human-in-the-loop hybrid augmented intelligence method

To validate the proposed human-in-the-loop hybrid augmented intelligence method, compare indicators related to efficiency and safety margins against those of human and machine intelligence. Different indicators are valued in various practical scenarios. Indicators from the “Reject-priority” strategy method are suitable for scenarios prioritizing safety, while those from the “Clear-priority” strategy method are suited for scenarios emphasizing efficiency.

1. Performance of the method based on the “Reject-priority” strategy

Following the procedure outlined in Section 3.2.1, the contraband detection model initially processed all images in the dataset to make decisions. Consequently, the dataset was divided into two subsets: “Machine-reject” and “Machine-clear.” Subsequently, images from the “Machine-clear” subset were sent to human decision-making review. As a result, the subsets were reorganized into “Hybrid-reject” and “Hybrid-clear,” as depicted in Table 5.

**Table 5:** Subset classification for validation of the “Reject-priority” strategy method.

Duration (min)	7,328				
Numbers of images	33,819				
Machine decision subset	(Machine-reject) 13,240				(Machine-clear) 20,579
Human decision subset	(Hybrid-reject) 14,324				(Hybrid-clear) 19,495
Proportion of subset (%)	42.4				57.6
Case number	Rp-1	Rp-2	Rp-3	Rp-4	Rp-5
Subsets of different hybrid-decision	Machine first made correct decision of “Reject”	Machine first made incorrect decision of “Reject”	Machine first made incorrect decision of “Clear,” and then human made correct decision of “Reject”	Machine first made correct decision of “Clear,” but then human made incorrect decision of “Reject”	Human and machine made consistent correct decision of “Clear”
Numbers of images in hybrid-decision subset	8,288	4,952	339	745	19,495

There were 14,324 images in the “Hybrid-reject” subset, and 19,495 images in the “Hybrid-clear” subset. The total number of images that should be made decision of “Reject” was the sum of cases “Rp-1” and “Rp-3,” namely 8,288 + 339 = 8,627. The total number of images that should be made decision of “Clear” was the sum of cases “Rp-2,” “Rp-4” and “Rp-5,” namely 4,952 + 745 + 19,495 = 25,192.

- Indicators related to efficiency
  - The “Average time consuming per image” was (7,328*60)/(33,819 + 20,579) = 8.1 s
  - The “Rate of making ‘Clear’ decision” was 19,495/33819*100% = 57.6%
  - The “Contraband detection precision” was (8,288 + 339)/14324*100% = 60.2%
  - The “False alarm rate” was (4,952 + 745)/14324*100% = 39.8%
- Indicators related to safe margin
  - The “Contraband recall rate” was (8,288 + 339)/8,627*100% = 100%
  - The “Contraband missed detection rate” was 0/8,627*100% = 0%

1. Performance of the method based on the “Clear-priority” strategy.

According to the procedure detailed in Section 3.2.2, after the initial phase, images from the “Machine-reject” subset were forwarded to the human decision-making review. Consequently, the subsets were reorganized into “Hybrid-reject” and “Hybrid-clear,” as illustrated in Table 6.

**Table 6:** Performance of method based on “Clear-priority” strategy.

Duration (min)	4,734				
Numbers of images	33,819				
Machine decision subset	(Machine-reject) 13,240		(Machine-clear) 20,579		
Human decision subset	(Hybrid-reject) 8,767		(Hybrid-clear) 25,052		
Proportion of subset (%)	25.9		74.1		
Case number	Cp-1	Cp2	Cp-3	Cp-4	Cp-5
Subsets of different hybrid-decision	Human and machine made consistent correct decision of “Reject”	Human and machine made consistent incorrect decision of “Reject”	Machine first made incorrect decision of “Clear”	Machine first made correct decision of “Clear”	Machine first made incorrect decision of “Reject,” and then human made correct decision of “Clear”
Numbers of images in hybrid-decision subset	8,288	479	339	20,240	4,473

There were 8,767 images in the “Hybrid-reject” subset, and 25,052 images in the “Hybrid-clear” subset. The total number of images that should be made decision of “Reject” was the sum of cases “Cp-1” and “Cp-3,” namely 8,288 + 339 = 8,627. The total number of images that should be made decision of “Clear” was the sum of cases “Cp-2,” “Cp-4” and “Cp-5,” namely 479 + 20,240 + 4,473 = 25,192.

- Indicators related to efficiency
  - The “Average time consuming per image” was (4,734*60)/(33,819 + 13,240) = 6.0 s
  - The “Rate of making ‘Clear’ decision” was 25,052/33819*100% = 74.1%
  - The “Contraband detection precision” was 8288/8767*100% = 94.5%
  - The “False alarm rate” was 479/8767*100% = 5.5%
- Indicators related to safe margin
  - The “Contraband recall rate” was 8288/8627*100% = 96.1%
  - The “Contraband missed detection rate” was 339/8627*100% = 3.9%

1. General comparison of performances between different decision-making methods

It is recommended that a human-machine hybrid decision-making method can compensate for the weaknesses inherent in both human and machine intelligence. This method is not only more efficient but also more reliable. The indicators related to efficiency, safety margins and staff workload are summarized in Table 7.

**Table 7:** Comparison of performances between different decision-making methods.

Performance		Data resource			
Classification	Indicator	Human decision-making	Machine decision-making	Hybrid decision-making	
				Reject-priority	Clear-priority
Efficiency	Average time consuming per image(s)	21.2	0.1	8.1	6.0
	Rate of making “Clear” decision (%)	73.4	60.9	57.6	74.1
	Contraband detection precision (%)	86.4	62.6	60.2	94.5
	False alarm rate (%)	13.6	37.4	39.8	5.5
Safe margin	Contraband recall rate (%)	90.3	96.1	100	96.1
	Contraband missed detection rate (%)	9.7	3.9	0	3.9
Staff workload	Total amount of images for human inspecting (images)	33,819	0	20,579	13,240

The hybrid decision-making method based on both strategies significantly reduces “average time consuming per image” and “staff workload” compared to human decision-making across all indicators. For other metrics, the performance varies depending on the underlying strategy. Specifically, the “Hybrid decision-making based on ‘Reject-priority’” strategy yields “False alarm rate” and “Contraband recall rate,” but lower “Rate of making ‘Clear’ decision,” “Contraband detection precision” and “Contraband missed detection rate.” Conversely, the “Hybrid decision-making based on ‘Clear-priority’ strategy” exhibits improved “Rate of making ‘Clear’ decision,” “Contraband detection precision” and “Contraband recall rate,” along with reduced “False alarm rate” and “Contraband missed detection rate.”

The proposed hybrid decision-making method exhibits unique characteristics based on each strategy. While the “Reject-priority” method excels in safety margin related indicators, its efficiency is suboptimal. Conversely, the “Clear-priority” method offers a more balanced distribution of indicators across various metrics.

## 5 Discussion

To evaluate the practical value for security inspections, we employ five attributes for a comprehensive assessment of the decision-making method outlined earlier. Certain indicators in Table 7 correspond to specific attributes of the “decision-making” method, which is further elaborated upon in Table 8.

**Table 8:** Correlations between “Attributes” and “Indicators.”

Attributes	Corresponding Indicator	Correlation
Speed of decision-making	Average time consuming per image	Negative
Ability of making correct decision	Rate of making “Clear” decision	Non-linear
Capability of anti-interference	False alarm rate	Negative
Ability of security risk perception	Contraband recall rate	Positive
Ability of reducing labor intensity	The total amount of images for human inspecting	Positive

**Figure 6:**
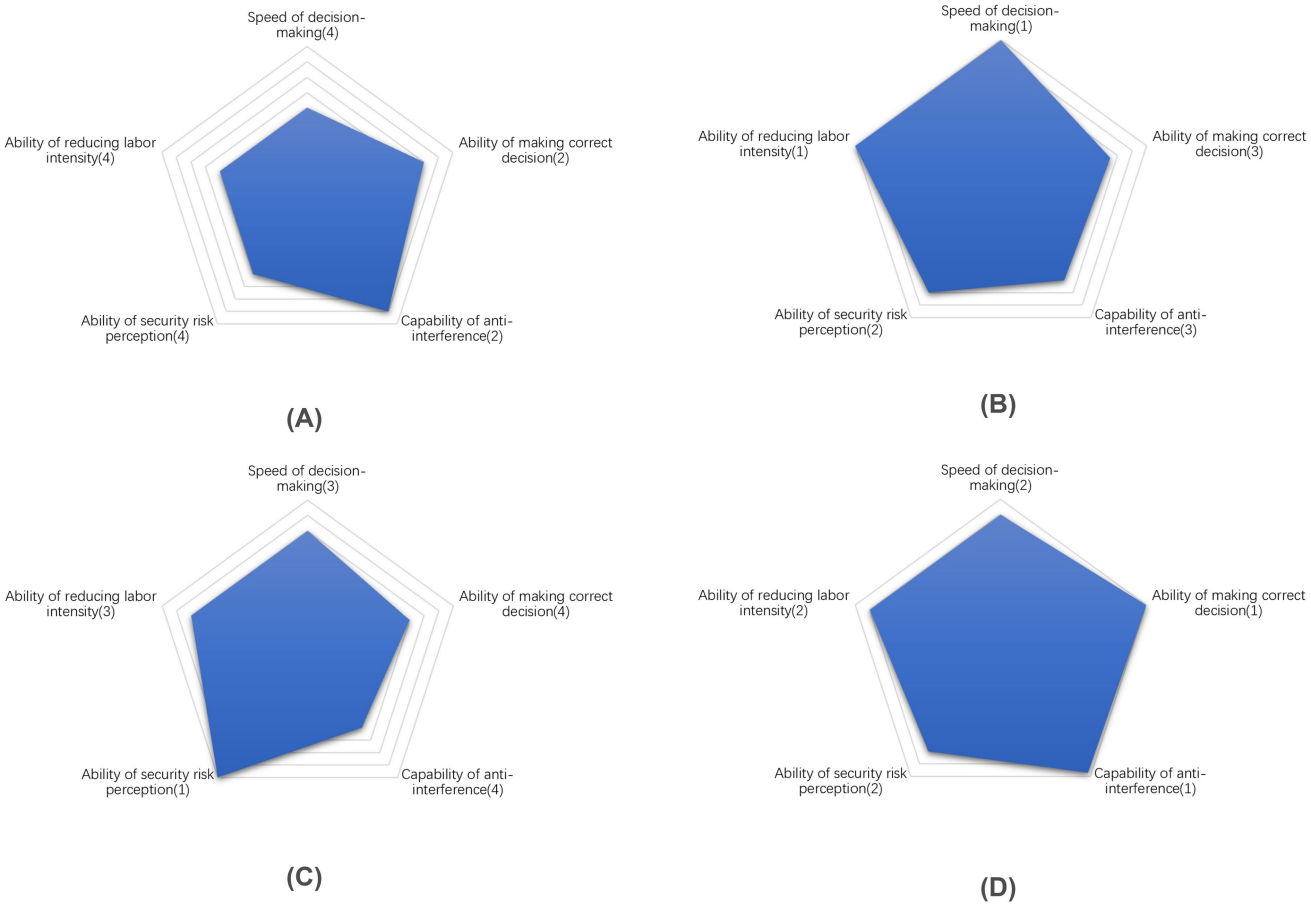
Performance comparison on five aspects for each method. **(A)** Human decision-making. **(B)** Machine decision-making. **(C)** Hybrid decision-making (Reject-first). **(D)** Hybrid decision-making (Clear-first).

Using the attributes in Table 8 as the axis, a “Radar chart” is created for each decision-making method, as depicted in Figure 6. Each coordinate point’s value is derived from sorting the corresponding indicator’s value for that particular method. The specific sorting rules are based on the deviation of the indicator’s actual value from its theoretical value. Indicators positively correlated with the attributes receive higher rankings for higher numerical values, while those negatively correlated receive higher rankings for lower numerical values. All the above values are summarized in Table 9.

**Table 9:** “Radar chart” coordinate value overview.

Indicator	Theoretical ideal value	Deviation from ideal value (rank)			
		Human decision-making	Machine decision-making	Hybrid decision-making	
				Reject-priority	Clear-priority
Average time consuming per image(s)	→0	21.2 (4)	0.1 (1)	8.1 (3)	6.0 (2)
Rate of making “Clear” decision (%)	74.5 (25,192/33,819)	1.1 (2)	13.6 (3)	16.9 (4)	0.4 (1)
False alarm rate (%)	0	13.6 (2)	37.4 (3)	39.8 (4)	5.5 (1)
Contraband recall rate (%)	100	9.7 (4)	3.9 (2)	0 (1)	3.9 (2)
Total amount of images for human inspecting (images)	0	33,819 (4)	0 (1)	20,579 (3)	13,240 (2)

Figure 6A illustrates the “Human decision-making” method, which excels in anti-interference and reliability in decision-making processes. However, this approach comes at the cost of human labor and the efficiency reduction. Furthermore, human error contributes to a relatively lower recall rate of contraband, thereby impacting the ability to perceive security risks effectively.

Figure 6B demonstrates that the speed of the “Machine decision-making” process is the fastest, completely liberating human labor. This contrasts sharply with the traditional “Human decision-making” approach. While the latter exhibits strong security risk perception capabilities, it remains sensitive to interference, resulting in frequent interruptions.

The characteristics of the “Hybrid decision-making based on ‘Reject-priority’” method are illustrated in Figure 6C. This approach exhibits several notable advantages in security risk perception:

- It integrates the contraband detection abilities of both humans and machines.
- On the proposed dataset, the recall rate for contraband achieves 100%.

However, there are significant drawbacks to this method:

- Its ultimate performance in security risk perception comes at a substantial cost.
- The system is highly sensitive to interference, making it less robust.
- The overall performance of the method is relatively conservative.

This conservatism translates to an extremely cautious approach in security inspection scenarios with high safety standards. While this ensures maximum safety, it may also lead to unnecessary delays or false positives in certain situations.

The “Hybrid decision-making based on ‘Clear-priority’” method illustrated in Figure 6D exhibits superior and well-balanced performance. This approach not only boasts an almost flawless capacity for accurate decision-making but also significantly reduces dependence on human labor. Furthermore, it achieves comparable performance in security risk perception to machine-based decision-making while demonstrating enhanced resistance to interference. In essence, this hybrid method addresses the limitations of both human and machine approaches, effectively combining their respective strengths. It is particularly well-suited for security inspection scenarios that demand high timeliness and handle substantial volumes of transactions.

Comparing the actual effects of the two strategies, it can be seen that the “Reject-priority” strategy has reached the ultimate level in the “Safe Margin,” but at the expense of efficiency. While it may cause some inconvenience to passengers from the perspective of security service experience. However, it appropriately reduces the workload of security inspectors.

The “Reject-priority” strategy is particularly suitable for security scenarios with high safety standards, such as: Important security venue inspections, Major event inspections, Civil aviation passenger inspections and Border checkpoint inspections.

On the other hand, the “Clear-Priority” strategy has been significantly optimized in terms of efficiency and labor intensity. It has also been optimized to a certain extent in the “Safety Margin” indicator. This strategy is well-suited for security scenarios with high requirements for inspection timeliness and large business flow throughput, such as: Express logistics inspections, Urban rail transit inspections during peak periods, Railway inspections during the Spring (Summer) transportation period.

Certainly, there are still some limitations in the practical implementation of the security screening system based on human-machine hybrid decision-making. The “Reject-priority” strategy is relative sensitive, resulting in a higher “Reject rate,” which causes inconvenience to the inspected individuals and leads to a decrease in system usability. Additionally, while the “Clear-priority” strategy greatly reduces the workload of security inspector, it may lead to decreased vigilance and over-reliance on machines, potentially resulting in vulnerabilities in identifying rare contraband.

## 6 Conclusion

In this paper, we propose a human-in-the-loop hybrid augmented intelligence approach tailored to address the practical requirements of security inspection systems. This methodology manifests itself as a hybrid decision-making method that incorporates two distinct strategies: “Reject-priority” and “Clear-priority.” These strategies serve complementary roles in enhancing the overall performance of the decision-making process.

On the dataset from a specific practical security inspection site, comparative experiments were conducted to validate the performance of the hybrid decision-making method. Initially, the differences of capabilities between human inspector and machine intelligence were compared across some indicators. Several findings emerged, revealing that machine decision-making exhibits higher safety margins and efficiency but lower confidence in decision-making compared to human decision-making. Consequently, a further validation with more indicators of performance metrics was employed to validate the proposed “Hybrid decision-making” method. Subsequently, evaluations are transformed into a side-by-side comparison of relative differences in terms of multidimensional capabilities among “Human,” “Machine,” and “Hybrid” decision-making method. Based on these comparisons, several conclusions were drawn.

1. “Hybrid decision-making” method based on strategies both exhibit superior “contraband recall rates” compared to traditional “Human decision-making.” This enhanced capability for risk perception contributes to a broader safety margin in the security inspection system.
2. “Hybrid decision-making” method based on strategies both can reduce human labor requirements compared to “Human decision-making.” This leads to increased efficiency and lower labor cost for the security inspection system.
3. “Hybrid decision-making” method based on strategies both methods are less time-consuming than “Human decision-making” resulting in a higher speed of decision-making that improves the overall efficiency of the security inspection system.
4. “Hybrid decision-making based on ‘Clear-priority’” has more balanced performance than “Hybrid decision-making based on ‘Reject-priority.’” It complements the strengths of humans and machines separately while reflecting both simultaneously. In comparison, “Hybrid decision-making based on ‘Reject- priority’” demonstrates superior performance in risk perception, integrating the contraband detection capabilities of both humans and machines.
5. Hybrid decision-making method with basis of either strategy can enhance decision-making effectiveness by integrating human and machine intelligence. Various strategies support different levels of method enhancement. Each method is tailored to specific practical scenarios based on its characteristics. “Reject-priority” is particularly suitable for security inspection scenarios requiring stringent safety protocols, such as: security checks at critical facilities, major event security screenings, civil aviation passenger security inspections, border checkpoint security screenings. “Clear-priority” is ideal for security inspection scenarios characterized by high-volume traffic flow and demanding efficiency, including express luggage security screening, peak-hour security checks in urban rail transit systems, large-scale event security screenings.

In the future, research will continue on how to fuse multi-hybrid intelligence strategies based on dynamic allocation of efficiency and workload. The goal is to select the optimal strategy considering instantaneous traffic flow in practical sites. Furthermore, as the scale of collected data continues to grow, the approach will enable seamless adaptation of the method to the actual needs of the application.

Simultaneously, the results of the security inspector not only allow for correcting incorrect machine decisions but also facilitate the collection of machine incremental learning samples spontaneously. These samples serve as a data source for the self-evolution machine intelligence. As machine capabilities continue to advance, the scope of work that can be automated increase gradually.

This method systematically enhances the effectiveness of security inspections while reducing labor costs. By optimizing both efficiency and safety margins, it provides a comprehensive solution for various security scenarios, ranging from high-safety standard venues to high-throughput situations requiring rapid processing.

## Data Availability

The datasets presented in this article are not readily available because the data is confidential to the Institute. Requests to access the datasets should be directed to HongJi Zhang 962051393@qq.com.
